# Percutaneous pedicle screw reduction and axial presacral lumbar interbody fusion for treatment of lumbosacral spondylolisthesis: A case series

**DOI:** 10.1186/1752-1947-5-454

**Published:** 2011-09-12

**Authors:** Gabriel C Tender, Larry E Miller, Jon E Block

**Affiliations:** 1 Department of Neurosurgery, Louisiana State University School of Medicine, 2020 Gravier Street, Room 336A, New Orleans, LA 70112, USA; 2 Miller Scientific Consulting, Inc., 422 Mountain Wasp Drive, Biltmore Lake, NC 28715, USA; 3 Jon E. Block, PhD, Inc., 2210 Jackson Street, Suite 401, San Francisco, CA 94115, USA

## Abstract

**Introduction:**

Traditional surgical management of lumbosacral spondylolisthesis is technically challenging and is associated with significant complications. The advent of minimally invasive surgical techniques offers patients treatment alternatives with lower operative morbidity risk. The combination of percutaneous pedicle screw reduction and an axial presacral approach for lumbosacral discectomy and fusion offers an alternative procedure for the surgical management of low-grade lumbosacral spondylolisthesis.

**Case presentation:**

Three patients who had L5-S1 grade 2 spondylolisthesis and who presented with axial pain and lumbar radiculopathy were treated with a minimally invasive surgical technique. The patients-a 51-year-old woman and two men (ages 46 and 50)-were Caucasian. Under fluoroscopic guidance, spondylolisthesis was reduced with a percutaneous pedicle screw system, resulting in interspace distraction. Then, an axial presacral approach with the AxiaLIF System (TranS1, Inc., Wilmington, NC, USA) was used to perform the discectomy and anterior fixation. Once the axial rod was engaged in the L5 vertebral body, further distraction of the spinal interspace was made possible by partially loosening the pedicle screw caps, advancing the AxiaLIF rod to its final position in the vertebrae, and retightening the screw caps. The operative time ranged from 173 to 323 minutes, and blood loss was minimal (50 mL). Indirect foraminal decompression and adequate fixation were achieved in all cases. All patients were ambulatory after surgery and reported relief from pain and resolution of radicular symptoms. No perioperative complications were reported, and patients were discharged in two to three days. Fusion was demonstrated radiographically in all patients at one-year follow-up.

**Conclusions:**

Percutaneous pedicle screw reduction combined with axial presacral lumbar interbody fusion offers a promising and minimally invasive alternative for the management of lumbosacral spondylolisthesis.

## Introduction

Patients with intractable low back pain or radiculopathy (or both) resulting from lumbar or lumbosacral spondylolisthesis benefit from surgical intervention [1,2]. Standard surgical protocols use a midline incision, posterior decompressive laminectomy, and posterolateral or interbody fusion (or both) [1]. Minimally invasive spinal surgery techniques have recently allowed the surgeon to obtain comparable clinical and radiographic results with less iatrogenic soft tissue injury and minimal blood loss. These techniques use a tubular retractor and the transforaminal approach, usually augmented with percutaneous placement of pedicle screw systems [3,4]. The recently developed AxiaLIF System (TranS1, Inc., Wilmington, NC, USA) uses the presacral 'safe zone' to provide access to the L5-S1 or L4-5, L5-S1 interspaces for discectomy and fusion and achieves fusion rates similar to those of the transforaminal approach but with less risk of nerve injury [5]. The purpose of this case series was to describe an alternative technique for the treatment of lumbosacral spondylolisthesis. This alternative combines a percutaneous pedicle screw reduction system and the AxiaLIF technique.

## Case presentation

**Figure 1:**
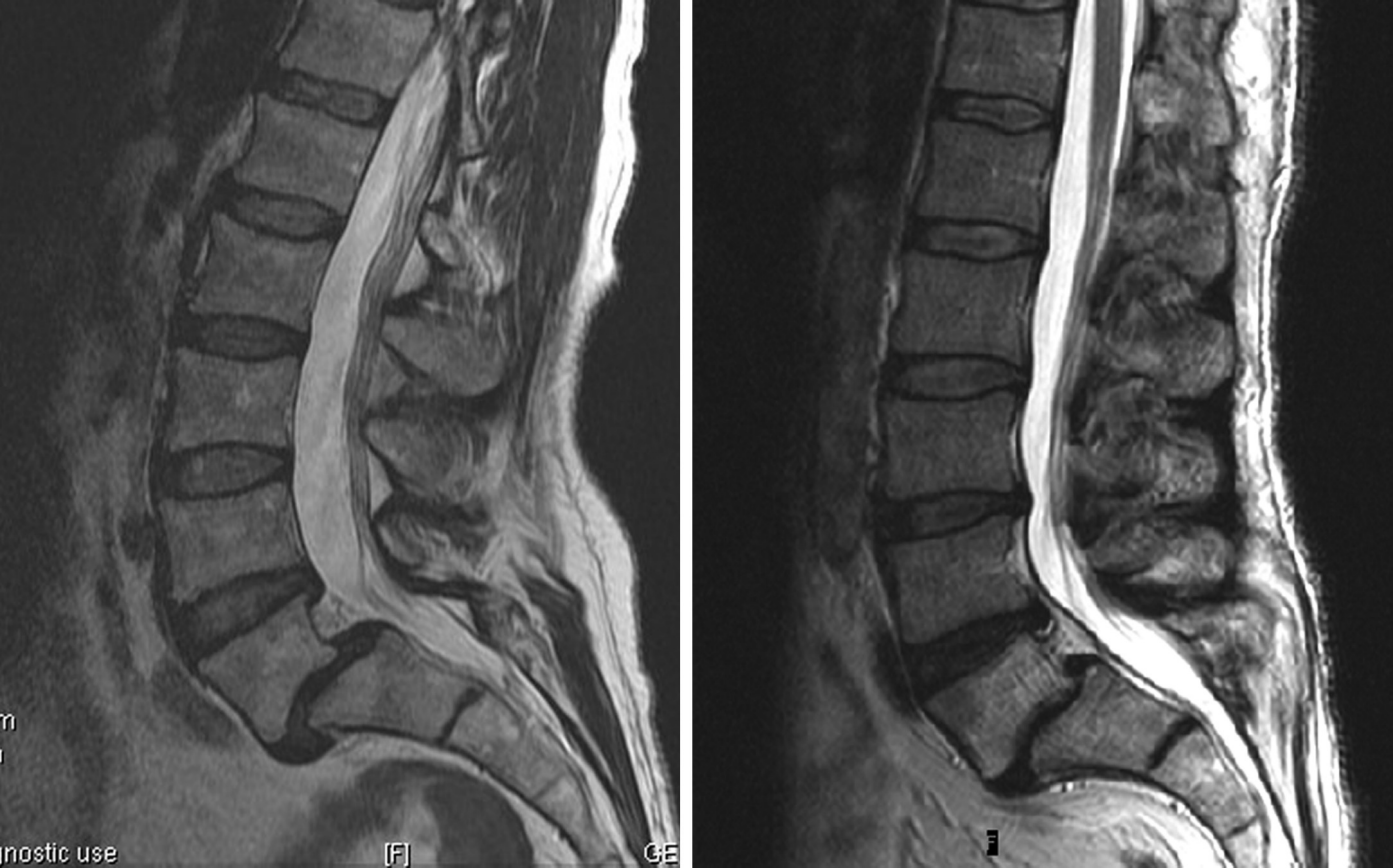
**Lumbar midline sagittal T2-weighted magnetic resonance images**. Grade 2 L5-S1 spondylolisthesis (case 1, left) and combined grade 2 L5-S1 anterolisthesis and grade 1 L4-5 retrolisthesis (case 3, right) are shown.

Three patients who underwent this operative procedure were evaluated retrospectively. The operations were performed between September 2009 and February 2010 at the Louisiana State University academic hospital (New Orleans, LA, USA). Preoperative imaging included lumbosacral magnetic resonance imaging (MRI) to assess the diseased segments (Figure 1) and to evaluate potential contraindications for the presacral approach (for example, hooked or flat sacrum, large crossing vessels, or minimal presacral fat). Flexion-extension lateral X-rays were used to evaluate spondylolisthesis mobility. The patients were selected on the basis of imaging evidence of low-grade spondylolisthesis and axial pain with concomitant lumbar radiculopathy [6]. Preoperative lumbar MRI demonstrated grade 2 spondylolisthesis at L5-S1 in all patients and an associated grade 1 spondylolisthesis at L4-L5 in one case.

A standardized surgical protocol was used for each case. Each patient was placed prone on a translucent operative table on a Wilson frame (to allow the initial exploration of the presacral space) with ample space for the C-arms underneath the table at the level of the lumbar and sacral spine. Biplanar fluoroscopy was used throughout each case.

The procedure began with the percutaneous pedicle screw placement as previously described [4]. Briefly, two 2 cm incisions were made, and each was about 4 cm on either side of the midline and centered over the disc space of interest. Next, a Jamshidi needle was docked at the junction between the transverse process and lateral facet and then advanced in a lateral-to-medial direction. When the tip of the needle reached the base of the pedicle on the lateral image, the anteroposterior image showed the tip within the oval shape of the pedicle but not past its medial border. Neurostimulation of the needle was performed at this time to confirm that the wall of the pedicle was not breached, and this was followed by a K-wire, tap, and pedicle screw. This procedure was repeated for the other pedicles; the only difference was that a Ferguson modification of the anteroposterior fluoroscopic view was used for the S1 pedicle cannulation.

**Figure 2:**
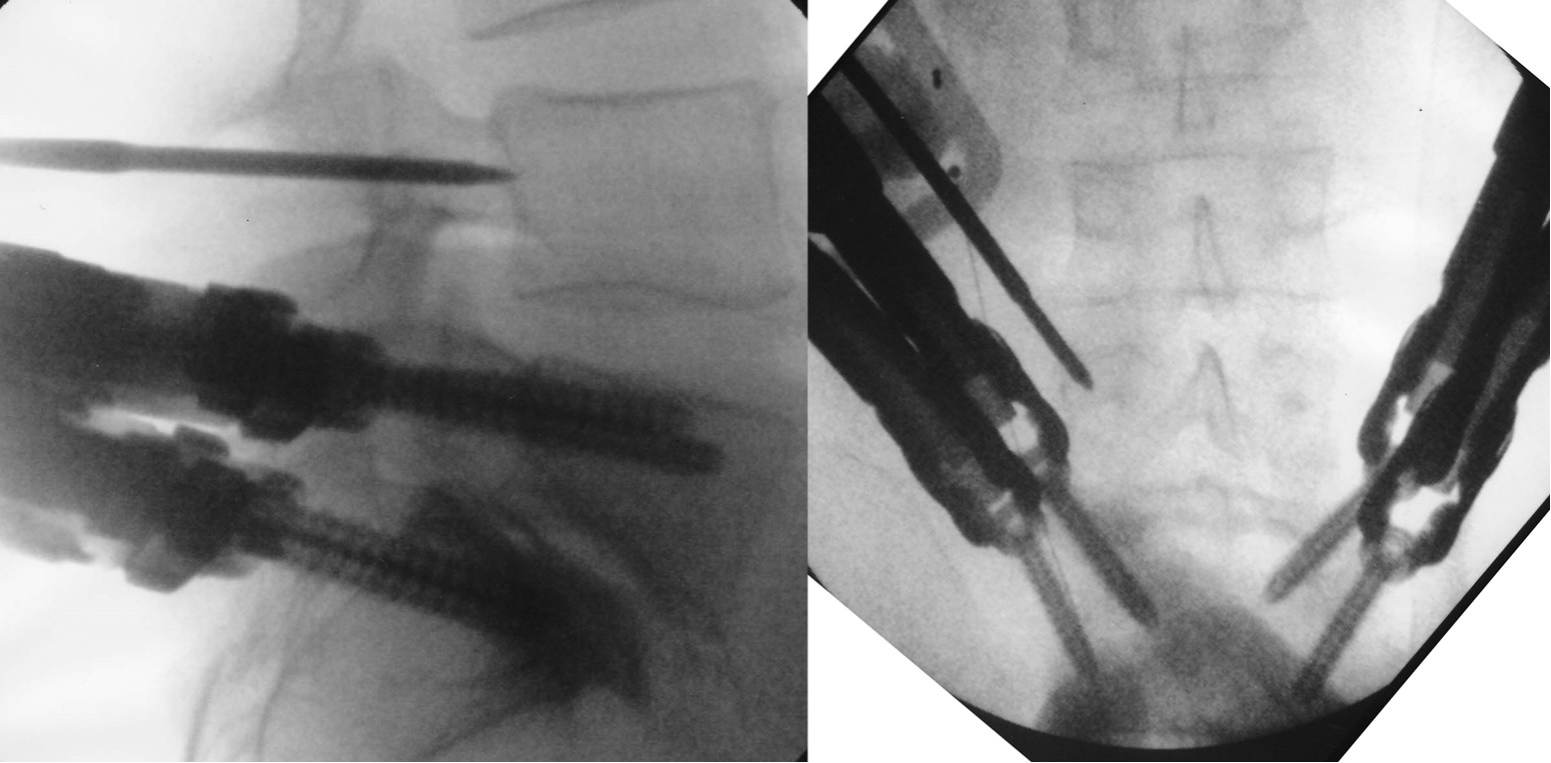
**Intraoperative lateral (left) and anteroposterior (right) fluoroscopic images depict Jamshidi needle insertion into the left L4 pedicle (case 3)**. When the tip of the needle reaches the base of the pedicle on the lateral image, it remains within the pedicle contour on the anteroposterior image.

We used the CD Horizon Sextant system (Medtronic Sofamor Danek, Memphis, TN, USA) for percutaneous reduction of lumbar spondylolisthesis as previously described [4]. For the patient with L4-5 and L5-S1 disease, the screws at L5 and S1 were initially placed and the trajectory for the L4 pedicle screw was determined to accommodate the percutaneously inserted rod (Figure 2).

The reduction screw extender allowed up to 2 cm of translation of the listhesed vertebral body relative to the extender body, hence the limitation of this technique to spondylolisthesis cases of not more than grade 2. Long screws with good purchase were used for the L5 pedicle since high pull-out strength is essential for successful reduction. Once spondylolisthesis reduction was achieved, the screw caps were attached and temporarily locked in place. Attention was given to the presacral approach.

The trajectory of the axial rod was planned on sagittal MRI images before the operation. With the spondylolisthesis at least partially reduced, this anterior trajectory was deemed feasible in all cases on the basis of preoperative imaging analysis. Therefore, a 2 cm paracoccygeal skin incision was made and the presacral approach was performed to place the anterior axial rod as previously described [7]. Specifically, the entry point was selected under fluoroscopic guidance close to the S1-2 junction (on the lateral images) and close to the midline (on the anteroposterior images) so that the extension of a straight line from the entry point would cross the center of the L5-S1 disc (for the single-level cases) or the center of the L5 vertebral body (for the two-level case). The trajectory was further adjusted as the guide pin or hand drill or both were advanced through the bone by turning the bevel of the guide pin in the desired direction or controlling the back of the hand drill.

**Figure 3:**
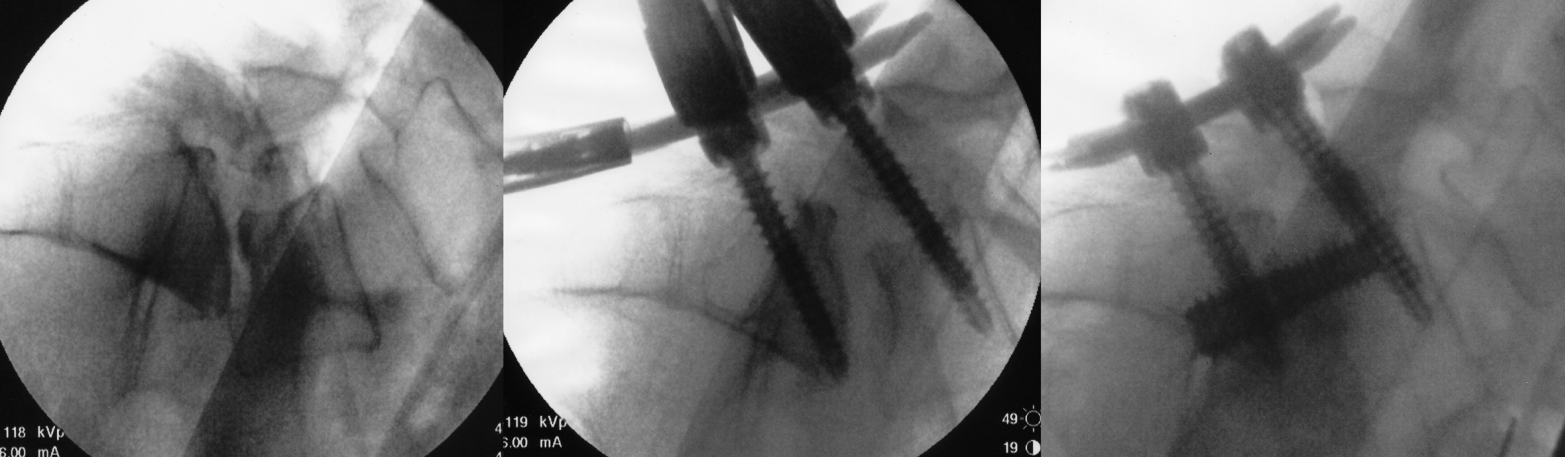
**Intraoperative lateral fluoroscopic images of L5-S1 spondylolisthesis reduction and distraction (case 1)**. The grade 2 spondylolisthesis (left) is reduced to grade 1 by using the percutaneous pedicle screws (middle), and the L5-S1 interspace is further distracted by using the anterior axial rod.

**Figure 4:**
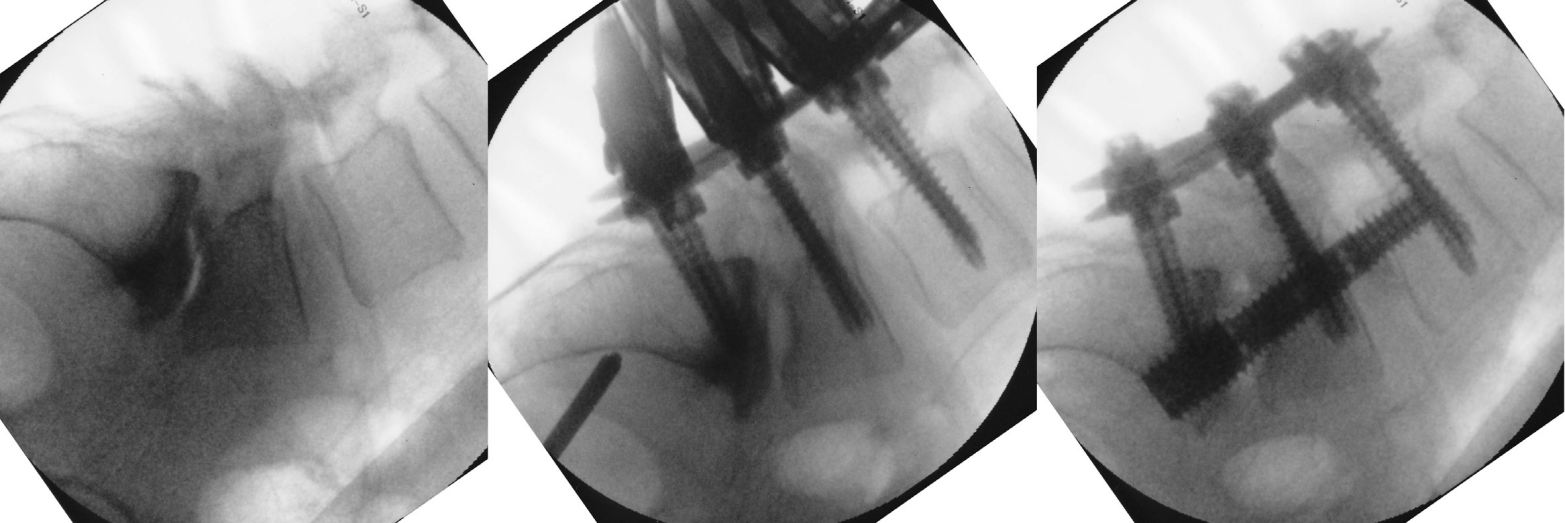
**Intraoperative lateral fluoroscopic images of grade 2 L5-S1 anterolisthesis and grade 1 L4-5 retrolisthesis reduction and distraction (case 3)**. The spondylolisthesis (left) is reduced by using the percutaneous pedicle screws (middle), and the L5-S1 interspace is further distracted by using the two-level anterior axial rod.

Initially, a volumetric discectomy was performed by using specially designed cutting-loop devices and disc extractors. Next, bone graft (a mixture of INFUSE recombinant human bone morphogenetic protein-2 [Medtronic Sofamor Danek], tricalcium phosphate, and autograft harvested during the trajectory creation) was inserted to promote interbody fusion. The threaded axial rod was advanced along the guide pin through S1 and into the L5 vertebral body. The superior aspect of this threaded rod, designed to engage the L5 vertebral body, had a wider thread pitch than the inferior S1 portion of the device, allowing intervertebral distraction by a reverse lag-type screw action. Once the axial rod was engaged into L5, the pedicle screw caps were partially loosened, and the axial rod was used to prevent loss of reduction. The rod was further advanced into the L5 vertebral body. By anchoring the rod and releasing the posterior percutaneous pedicle screw caps, distraction of the involved interspace combined with maintenance of reduction was achieved. By design, the axial rod can provide minimal (1 to 2 mm), medium (2 to 4 mm), or maximum (4 to 6 mm) distraction of the spinal interspace upon insertion. We used either minimal or medium distraction axial rods to further indirectly decompress the neural foramina (Figure 3). The procedure was extended in a similar fashion to L4 in one patient with L4-5 and L5-S1 disease (Figure 4). Once the axial rod was advanced to its final position, the pedicle screw caps were tightened and the three small wounds were closed in layers.

**Figure 5:**
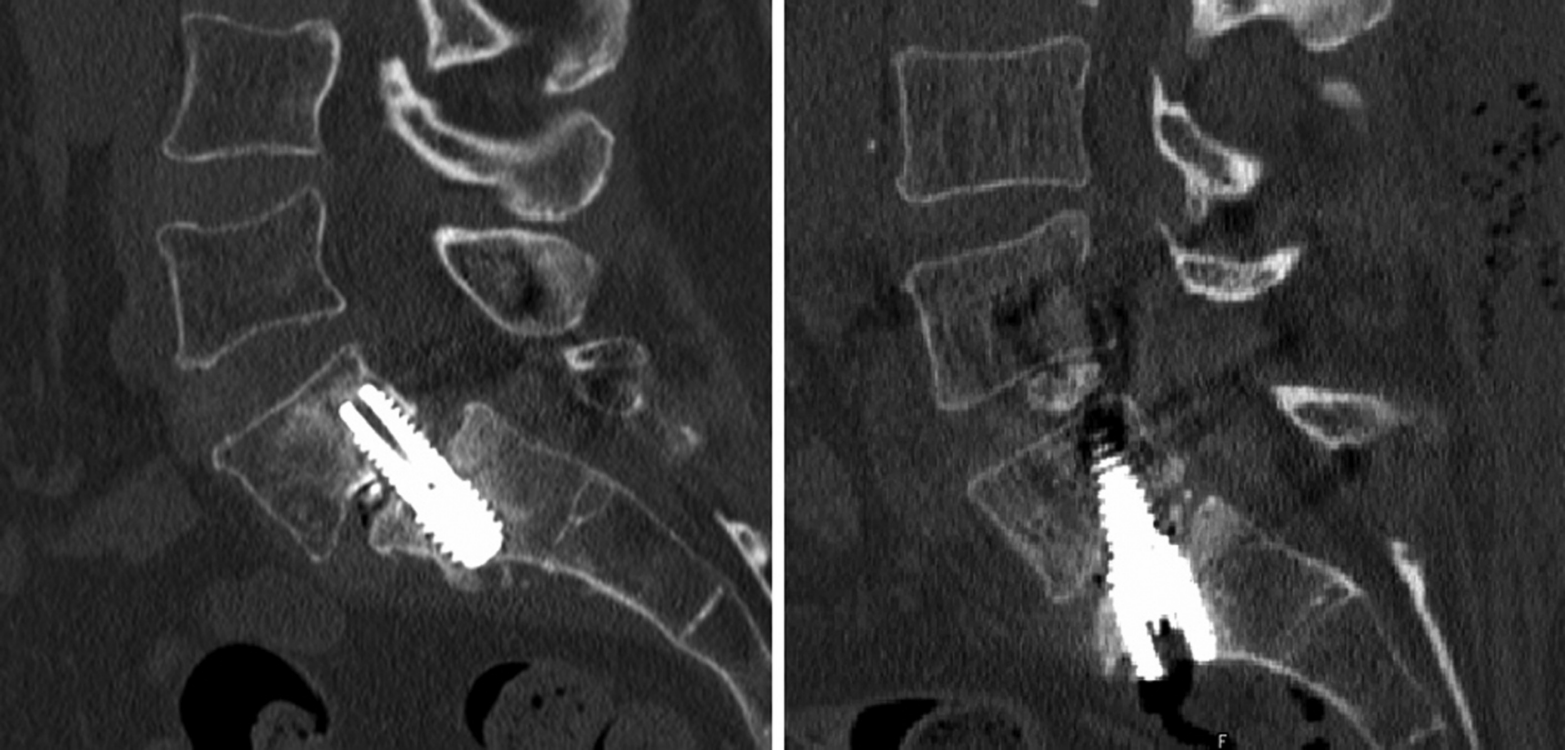
**Computed tomographic midline sagittal images of the lumbosacral spine demonstrate postoperative spinal alignment for L5-S1 (case 1, left) and L4-5, L5-S1 (case 3, right) constructs**.

**Figure 6:**
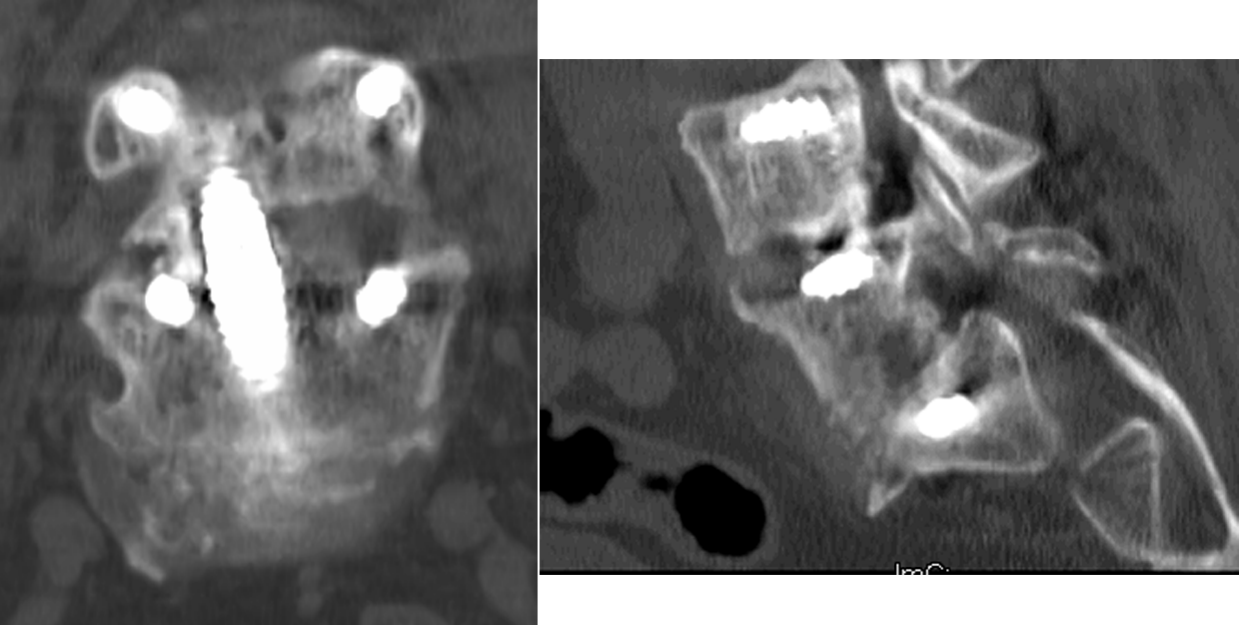
**Computed tomographic coronal (left) and sagittal (right) images demonstrate successful fusion at one-year follow-up**.

The first case was of a 46-year-old Caucasian man who presented with a four-year history of axial low back pain and radiculopathy that was described as 7 out of 10 on average and as 10 out of 10 at its worst on an 11-point Likert scale, of mechanical type (exacerbated by standing or walking for extended periods of time and improved by lying down), and refractory to extensive conservative treatment. During a physical examination, our patient showed no sensory-motor deficits. Lumbar MRI showed a grade 2 spondylolisthesis at L5-S1 with endplate Modic changes and severe bilateral foraminal stenosis. The operative time was 197 minutes, and blood loss was minimal (50 mL). Treatment with the AxiaLIF System reduced the spondylolisthesis to grade 1. Our patient was ambulatory after surgery and reported relief from back pain (maximal pain severity of 10 at pretreatment to 3 at post-treatment), resolution of radicular symptoms (10 to 1), improvements in back function (68% to 15% on the Oswestry Disability Index), and no complications. Our patient was discharged from the hospital two days after the procedure. To confirm the adequate placement of the instrumentation and to accurately evaluate the final constructs, a computed tomography scan was obtained after the operation (Figure 5). Wide indirect neuroforaminal decompression and solid fixation constructs were achieved. Successful fusion, defined as no motion at the treated segment on flexion/extension radiographs and evidence of bone growth between the adjacent vertebral bodies on reconstructed computed tomography images, was demonstrated at one-year follow-up (Figure 6).

The second case was a 51-year-old Caucasian female who presented with a 10-year history of axial low back pain and a one-year history of radiculopathy. The pain was described as 9 out of 10 on average and 10 out of 10 at its worst, of mechanical type, and refractory to conservative treatment. During a physical examination, our patient showed no sensory-motor deficits. Lumbar MRI showed a grade 2 spondylolisthesis at L5-S1 with severe bilateral foraminal stenosis. The operative time was 173 minutes, and blood loss was minimal (50 mL). Treatment with the AxiaLIF System reduced the spondylolisthesis to grade 0. Our patient was ambulatory after surgery and reported relief from back pain (10 to 2), resolution of radicular symptoms (10 to 1), improvements in back function (65% to 10%), and no complications. Our patient was discharged from the hospital two days after the procedure. Wide indirect neuroforaminal decompression and solid fixation constructs were achieved with successful fusion at one-year follow-up.

In the third case, a 50-year-old Caucasian man presented with an 18-year history of axial low back pain and a one-year history of radiculopathy. The pain was described as 8 out of 10 on average and 10 out of 10 at its worst, of mechanical type, and refractory to conservative treatment. During a physical examination, our patient showed no sensory-motor deficits. Lumbar MRI showed a grade 2 spondylolisthesis at L5-S1 with severe bilateral foraminal stenosis and a grade 1 spondylolisthesis at L4-5. The operative time was 323 minutes, and blood loss was minimal (50 mL). Treatment with the AxiaLIF System reduced the spondylolisthesis to grade 0 at L5-S1 and at L4-5. Our patient was ambulatory following surgery and reported relief from back pain (10 to 3), resolution of radicular symptoms (10 to 2), improvements in back function (69% to 14%), and no complications. Our patient was discharged from the hospital three days after the procedure. Wide indirect neuroforaminal decompression and solid fixation constructs were achieved with successful fusion at one-year follow-up.

## Discussion

Lumbosacral spondylolisthesis presents a challenge for the spine surgeon and is traditionally treated by either open anterior or posterior approaches. The surgical goals are to decompress the neural structures and to provide the appropriate environment for a solid fusion. The decompression, which is important for sagittal balance preservation [8], can be performed directly by removal of the lamina and pars interarticularis or indirectly by distraction of the spinal interspace or reduction of spondylolisthesis or both [9-11].

The advent of minimally invasive access techniques has revolutionized the field of spine surgery. The transforaminal approach followed by percutaneous pedicle screw reduction of spondylolisthesis has been described along with a report of acceptable clinical and radiographic results [4]. While this approach provides direct unilateral decompression of the neural foramen, access to the disc space is limited because of the steep angle and narrow working corridor inherent to spondylolisthesis cases. Severely collapsed disc spaces are frequently encountered in these patients, adding considerable technical challenges to achieving adequate interbody distraction through a minimally invasive approach.

In contrast, percutaneous reduction of spondylolisthesis by using bilateral pedicle screws offers several potential advantages. Reduction may be accomplished using simultaneous bilateral screw fixation, thus decreasing the risk of L5 pedicle screw pull-out and limiting the risk of endplate violation inherent to an initial interbody approach to reduction. Another major advantage of this technique is offered by the axial presacral approach, which allows anterior access to the L5-S1 (or L4-S1) discs in the prone position. Once the spondylolisthesis reduction is achieved with pedicle screws, placement of the anterior axial rod becomes routine and also offers the option of further indirect foraminal decompression. As before, this combined technique is possible only because both the percutaneous pedicle screw reduction and the presacral approach can be performed in the prone position.

One major concern with this surgical technique is the potential for the pedicle screw system to fail in reducing the spondylolisthesis. In this situation, the pedicle screws on one side may be temporarily removed and a minimally invasive transforaminal approach may be employed on that side through the same incision to provide the discectomy and interbody graft placement as previously described.

The AxiaLIF System is not intended to treat severe scoliosis, severe spondylolisthesis (grade 3 or 4), tumor, or trauma. Contraindications for use include coagulopathy, bowel disease, pregnancy, and sacral agenesis. Use of the AxiaLIF System is limited to anterior fusion of the lumbar spine at L5-S1 (2-LEVEL System for L4-S1) in conjunction with legally marketed posterior fixation systems. The AxiaLIF System should not be used with facet screws when spinal stenosis correction requires removal of significant portions of the lamina or any portion of the facets. The 2-LEVEL System is additionally contraindicated for patients with vertebral compression fractures or any other condition in which the mechanical integrity of the vertebral body is compromised.

Preoperative imaging should be thoroughly evaluated with emphasis on perirectal fat pad thickness, identification of the rectum/sacrum interface, aberrant vasculature, and anticipated trajectory. Thus, relative contraindications for the presacral approach include insufficient presacral fat pad, previously explored presacral space, large vessels crossing the presacral space, and anatomic abnormalities that preclude placement of an axial rod through the lower lumbar segments.

## Conclusions

This case series describes an alternative and viable approach for the treatment of lumbosacral spondylolisthesis. Spondylolisthesis reduction using a percutaneous pedicle screw system allows the placement of an anterior axial rod, which in turn can further distract the interspace and indirectly decompress the neuroforamina. This minimally invasive approach was used safely in three patients. A larger study with long-term follow-up is needed to validate this procedure.

## Abbreviations

MRI: magnetic resonance imaging.

## Consent

Written informed consent was obtained from the patients for publication of this case series and any accompanying images. A copy of the written consent is available for review by the Editor-in-Chief of this journal.

## Competing interests

The authors declare that they have no competing interests.

## Authors' contributions

GCT performed the surgeries, collected and interpreted patient data, and was involved in drafting the manuscript. LEM and JEB interpreted patient data and were involved in drafting the manuscript. All authors read and approved the final manuscript
